# The Role of Vitamin D in Cardiovascular Diseases

**DOI:** 10.3390/nu15163547

**Published:** 2023-08-11

**Authors:** Man Hung, Wendy C. Birmingham, Monica Ocampo, Amir Mohajeri

**Affiliations:** 1 College of Dental Medicine, Roseman University of Health Sciences, 10894 S. River Front Parkway, South Jordan, UT 84095, USA; 2 Department of Orthopaedic Surgery Operations, University of Utah, Salt Lake City, UT 84108, USA; 3 College of Social and Behavioral Sciences, University of Utah, Salt Lake City, UT 84112, USA; 4 Department of Psychology, Brigham Young University, Provo, UT 84602, USA

**Keywords:** vitamin D, cardiovascular disease, medicine, health

## Abstract

Cardiovascular diseases (CVD) are the leading cause of death in the United States. The previous literature demonstrates the importance of vitamin D for overall health, and a significant body of literature has examined the benefits of optimal serum 25-hydroxyvitamin D [25(OH)D] on cardiovascular health, but the results remain inconclusive. The objective of this study was to determine the association between reported CVD and [25(OH)D]. We utilized the 2015–2018 National Health and Nutrition Examination Survey and included adults aged 20 years and older (*n* = 9825). CVD was defined as having a stroke, heart attack, heart failure, or coronary heart disease. Vitamin D status was categorized as a serum 25(OH)D deficiency at <30 nmol/L; insufficiency at 30 to 49.9 nmol/L; normal/optimal at 50 to 125 nmol/L; and adequacy at >125 nmol/L. Statistical analysis was performed using Chi-square tests, *t*-tests were conducted to investigate the differences in participant characteristics among those with CVD and without CVD, and regression models were used to explore the association between vitamin D levels and CVD status. We found 25(OH)D deficiency associated with CVD (Adjusted Odds Ratio (AOR) = 1.48; 95% CI = 1.11–1.98; *p* < 0.05). [25(OH)D] insufficiency was also associated with CVD (AOR = 1.28; 95% CI = 1.06–1.54; *p* < 0.05). The 25(OH)D adequacy was not associated with reported CVD. For the prevention of CVD, healthcare professionals may recommend the use of vitamin D supplementation to improve cardiovascular health in adults while considering individual needs.

## 1. Introduction

Cardiovascular diseases (CVD) include coronary heart disease, rheumatic heart disease, heart attack, heart failure, cerebrovascular disease, and stroke. CVD are a major global health concern and the leading cause of death worldwide, with an estimated 17.9 million deaths each year, many of which are preventable [1], making it the most significant contributor to non-communicable disease mortality. In the United States (U.S.), heart disease is the leading cause of death for both men and women with 1 in 5 deaths in the U.S. in 2020 due to heart disease. This translates to one person dying every 34 s from heart disease, with an estimated cost of about USD 229 billion each year spent on healthcare services, medicines, and lost productivity [2,3,4]. While actual lifetime CVD risk is similar among men and women, sociodemographic differences contribute to greater CVD risk, poorer CVD outcomes, and mortality rates [5]. These differences include income, access to health care, education, insurance status, adverse childhood experiences, less social support, and reduced health literacy [6,7]. Race and ethnicity also play a role. Post et al., using data from the Multi-ethic Study of Atherosclerosis study, showed CVD mortality was higher in black participants than in white participants [6].

Although some contributing factors to CVD risk are not possible to modify, there are modifiable factors that can help prevent heart disease. These include eating a healthy diet, getting adequate and regular physical activity, and eliminating tobacco use. Metabolic factors such as increased body mass index, high cholesterol levels, and hypertension increase CVD risk [8] and may be lowered by a low-fat, high-fiber diet, aerobic exercise, and smoking cessation. Leafy green vegetables are particularly important for a healthy diet as these have significant benefits not just for CVD, but vegetable consumption has been shown to be inversely associated with the risk of developing hypertension and type 2 diabetes [9]. Leafy green vegetables have vascular benefits such as the conversion of inorganic nitrates to nitric oxide (NO) which can improve blood pressure and arterial stiffness.

Some modifiable factors are nonetheless unavailable for some individuals. Some do not or cannot maintain a healthy diet due to dietary restrictions or medical conditions. Vegans, breastfeeding women, illegal drug addicts, chronically ill people, or those with malabsorption problems (e.g., diarrhea, coeliac disease, and cystic fibrosis) may need supplements to improve vitamin and mineral deficiencies [10]. Others may not be able to obtain needed nutrients via diet alone due to socioeconomic (SES) factors such as low income, or living in lower SES neighborhoods in which there may be limited access to healthy fruits and vegetables. Individuals may thus take multivitamins to obtain needed nutrients and this can be beneficial in reducing risk for a variety of health conditions. For example, the recent systematic review by Sharadkumar [10] of multivitamin use found taking multivitamin supplements can promote brain function and support eye health. Yeung et al. found multivitamins improve memory recall [11] and Song et al. found those who take calcium or vitamin C supplements showed a lower risk of diabetes [12]. A large prospective study found multivitamin supplementation associated with a modest reduction in CVD [13] and in a recent examination of over 489,000 individuals from the NIH-American Association of Retired Persons (AARP), Lim and colleagues [14] found multivitamins to be cancer-preventive for colon cancer in both men and women, although they found no evidence of a protective role for other site-specific cancers. Gaziano and colleagues [15] also found that a daily multivitamin did not reduce site-specific cancers but was associated with reduced incidence of all cancers (excluding non-melanoma skin cancer). Fantacone et al. [16] found a statistically significant decrease in the severity and duration of reported illness, and Dudi [17] concluded that multivitamins can help heal psychiatric problems.

While the overall value of multivitamins can be seen in the literature, the role of vitamin D and its relationship to CVD remains unclear and is an important area of investigation, particularly given the prevalence of CVD. Vitamin D is synthesized from 7-dehydrocholesterol in the skin upon ultraviolet (UVB) light exposure from the sun. Circulating 25-hydroxyvitamin D [25-hydroxyvitamin D [25(OH)D] is the best indicator for determining vitamin D levels in the body [18]. Vitamin D plays a crucial role in various bodily systems, including the immune systems, cardiovascular systems [19], diseases and disorders such as rickets in children [20,21], and osteomalacia in adults [22]. Vitamin D has been shown to be necessary for optimal health [23] playing a role in the pathogenesis and course of osteoporosis, cancer progression and mortality, immune response, diabetes, hypertension, and CVD [19,24], although the literature on hypertension and CVD protection should be interpreted with caution. While research findings on the benefits of vitamin D in other health conditions are fairly consistent, the benefits of vitamin D for cardiovascular risk are mixed. A review of hundreds of studies found evidence that vitamin D is important for reducing the risk of a variety of chronic illnesses including cardiovascular disease [25]. Giovannucci et al. [26] found a nearly 2.5-fold increased risk of myocardial infarction (MI) for those with vitamin D deficiency in the Health Professionals Follow-up Study, and Acharya et al. found patients with no vitamin D deficiency and no history of MI or arterial fibrillation (AF) who were treated with (25-OH)D level of >20 ng/mL and >30 ng/mL showed significantly lower mortality risk and lower risk of AF [27,28]. Additionally, an analysis of individuals with resistant hypertension showed a significant association between resistant hypertension and vitamin D deficiency [29], and a meta-analysis by Mirhosseini, Rainsbury and Kimball [30] showed vitamin D supplementation associated with lower blood pressure, with an anti-inflammatory impact. Mirhosseini, Vatanparast and Kimball [31] found low 25(OH)D levels associated with higher systolic and diastolic blood pressure. Further, a recent examination of stroke risk by Wang, et al. [32] of 8523 participants in the NHANES found 25(OH)D deficiency a significant risk factor for stroke. Additional research has shown associations between vitamin D deficiency and left ventricular hypertrophy, endothelial dysfunction, hypertension, and arterial stiffness [33].

However, other studies have found no association between CVD prevention and vitamin D. The Women’s Health Initiative Calcium and Vitamin D Supplementation Study [34] and the Vitamin D Assessment Study [35] both showed vitamin D supplements had no effect on CVD risk. Additionally, a recent review by de la Guia-Galipienso and colleagues [36] concluded that vitamin D supplements did not show any appreciable benefits in reducing CVD risk even in the case of insufficiency. Further, in a 2023 assessment of findings regarding UV radiation, Neale et al. [37] noted there is evidence that UVA, and possibly UVB irradiation, influences blood pressure and CVD risk through the release of nitric oxide from stores in the skin rather than vitamin D itself. And a review by Scragg, Rahman and Thornley [38] found evidence that suggests sun exposure and UV exposure may have protective effects against blood pressure and CVD, although they submit that additional research is needed to determine if this is independent of vitamin D.

As previously mentioned, the main source of vitamin D is sunlight exposure, but such synthesis is highly variable and dependent on factors such as skin pigmentation, latitude, altitude, season of the year, and age. Additionally, UV radiation from the sun can cause significant damage to the skin, including skin cancer, changes to DNA, and immunosuppressive effects [39,40]. Thus, supplemental vitamin D is often used for those who show inadequate or deficient levels and supplemental use has increased over the past years, with up to a fourfold increase among U.S. adults from 1999–2012 [41]. These trends were seen across most age groups, races/ethnicities, and genders [42], although from 2013–2014 women, non-Hispanic whites, and those aged >70 were taking >4000 IU/day, which exceeds the tolerable limit and can lead to adverse effects [42].

Moreover, other work has found 25(OH)D > 125 nmol/L (adequacy) may actually increase the risk for CVD. Researchers found individuals were at increased risk for CV events and all-cause mortality at both deficiency and adequacy levels of 25(OH)D, although 25(OH)D deficiency still represents the greater risk [43,44]. In Durup and colleagues’ large observational study, they concluded that levels of 25(OH)D were associated with CVD diseases in a reverse J-shaped, with hazard ratios of 1.3 for CVD mortality for those with 25(OH)D adequacy. Dror et al. suggested a safe range of 20–36 ng/mL (50 to 90 nmol/L). A meta-analysis conducted by Zhang et al. found vitamin D levels between 40 to 50 ng/mL (i.e., 100 and 137 nmol/L) not related to CVD [45], while other studies have shown an increase in cardiovascular morbidity and all-cause mortality at vitamin D levels at or above 100 nmol/L [43,46,47,48].

These studies highlighted the discrepancies between the outcomes of various experimental studies and clinical intervention trials, indicating the need for further research in this area to fully understand the complex role of vitamin D in maintaining overall health and preventing disease.

In an effort to better understand and clarify the impact of vitamin D, and the specific levels of 25(OH)D on CVD risk, we collected data from 9825 participants enrolled in the NHANES, a large nationally representative sample, to conduct this study. Different societies and expert bodies have defined cutoffs that are generally the same [49]. We are using the cutoffs as defined by the Institute of Medicine [50], in which the optimal (i.e., normal) serum level of 25(OH)D concentration falls between 50 and 125 nmol/L (nanomoles per liter), with vitamin inadequacy between 30 and 49 nmol/L and vitamin deficiency as <30 nmol/L. Vitamin adequacy is defined as 25(OH)D >125 nmol/L.

## 2. Methods

### 2.1. Data Source

**Figure 1:**
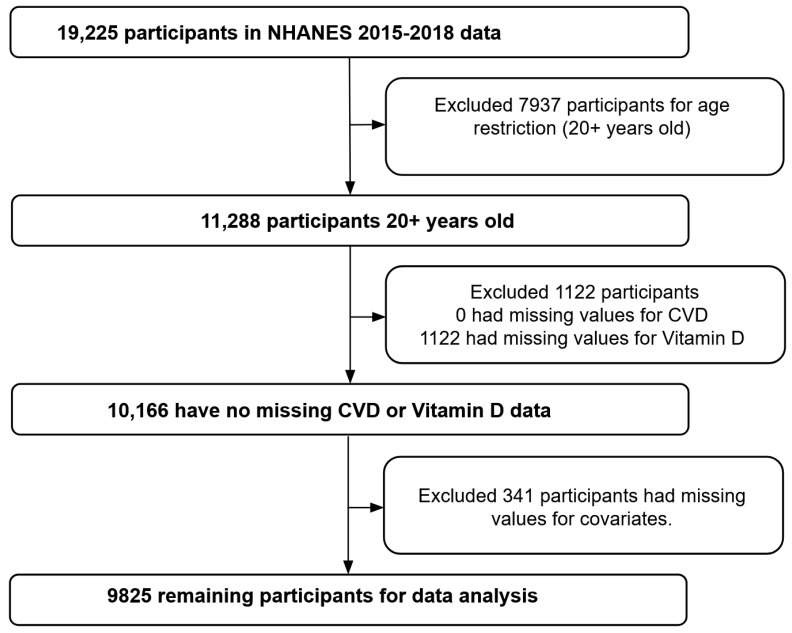
Flowchart displaying sample selection based on inclusion and exclusion criteria.

This study used the 2015 to 2018 data from the NHANES. Data collection for NHANES began in 1999 and were collected once every two years, with approximately 5000 individuals participating each year [51]. NHANES consists of both interviews and physical tests and uses complex sampling and weighting techniques to develop nationally representative estimates for assessing the health and nutrition status of the noninstitutionalized population in the U.S. Interviews were performed at the participant’s home, and health measurements were carried out in mobile centers across the U.S. For this study, data from two NHANES cycles (2015–2016 and 2017–2018) were combined for analysis in order to have a larger sample size and produce more stable estimates. Participants aged 20 years or older having CVD status data, vitamin D data, and relevant covariate data were included in the data analyses (Figure 1).

### 2.2. Measures

CVD status was defined as reporting a stroke, heart attack, heart failure, or coronary heart disease. In NHANES self-reported data, participants were considered to have a history of CVD if they responded “yes” to the question: “Has a doctor or other health professional ever told you that you had congestive heart failure or coronary heart disease or heart attack or stroke”?

Vitamin D was a continuous variable expressed as serum 25(OH)D in the NHANES database. The serum 25(OH)D level was measured using ultra-high-performance liquid chromatography–tandem mass spectrometry. Detailed measurement methods for serum 25(OH)D levels can be found in the NHANES procedure manual [52]. Serum 25(OH)D was categorized as recommended by the Institute of Medicine and noted above.

### 2.3. Covariates

Covariates such as demographic characteristics and risk factors that were associated with CVD were adjusted in the data analyses. These covariates included age, gender, race/ethnicity, body mass index (BMI), hypertension, hypercholesterolemia, diabetes, and C-reactive proteins (CRP) level. Age was measured at the time of the NHANES examination. Gender was classified as male and female; race/ethnicity was categorized as Hispanic, non-Hispanic white, non-Hispanic black, and other races. BMI was calculated by dividing weight in kilograms by height and measured in meters squared. The participant’s body measurements were collected at the mobile examination center (MEC) by trained health technicians. The height was measured with a stadiometer equipped with an adjustable headpiece and a fixed vertical backboard. A digital weight scale was used to weigh participants wearing the standard MEC examination gown. Participants were considered to have hypertension or hypercholesterolemia if they reported being told by a physician that they had high blood pressure or high blood cholesterol, respectively. Additionally, they were considered to have diabetes if they had been diagnosed by a physician, except when the diagnosis was made during their pregnancy. CRP was measured using latex-enhanced nephelometry, with a high CRP level defined as 0.20 mg/dL or greater.

### 2.4. Statistical Analysis

Descriptive statistics were calculated for the participants’ demographic characteristics and risk factors, 25(OH)D levels, and CVD status. Independent samples *t*-test and Chi-square test were used as appropriate to test the differences in participants’ characteristics by CVD status. Independent samples *t*-test was suitable for examining continuous variables across two different groups. The Chi-square test was applied to test two categorical variables. Two binary logistic regression models were used to examine the associations between 25(OH)D level and CVD status. We, therefore, reported adjusted odds ratio (AOR) and 95% confidence intervals (95% CI) as measures of association. To begin the regression analysis, we first modeled the association between 25(OH)D level and CVD status, adjusting for demographic factors (age, gender, and race/ethnicity) for Model 1. In Model 2, risk factors including BMI, hypertension, diabetes, hypercholesterolemia, and CRP were also controlled for, in addition to those factors used in Model 1. For all regression models, normal serum vitamin D levels were used as reference values. All analyses were conducted using SPSS software (SPSS version 28) and statistical significance was set at two-sided *p* < 0.05.

## 3. Results

### 3.1. Demographic Characteristics

The average age of the participants was 50.3 years old (interquartile range = 29.0 years) and the average BMI was 29.7 kg/m^2^ (interquartile range = 8.8 kg/m^2^). Most (52.1%; *n* = 5121) were female, and 34.0% (*n* = 3341) were non-Hispanic white. Most of the participants had never been told that they had high blood pressure (63.0%; *n* = 6194), diabetes (82.2%; *n* = 8075), high cholesterol (64.8%; *n* = 6369) or CVD (89.5%; *n* = 8798). Slightly over half (50.2%; *n* = 4937) of the participants had CRP levels below 0.2 mg/L, and 64.8% (*n* = 6370) had normal vitamin D levels (Table 1).

Table 2 shows the characteristics of the two sample groups: CVD participants and non-CVD participants. Individuals with CVD were significantly older, with more people who are male, non-Hispanic white, with a higher BMI, and other traditional factors than those individuals without CVD. The average BMI of CVD participants was 30.9 kg/m^2^ (interquartile range = 8.8 kg/m^2^) compared to 29.6 kg/m^2^ (interquartile range = 8.7 kg/m^2^) for non-CVD participants. There was also a greater proportion of people with high blood pressure, high cholesterol, diabetes, and CRP in the CVD group compared to the non-CVD group. Both groups had a predominantly normal 25(OH)D level (68.1% in the CVD participants and 64.5% in the non-CVD participants).

### 3.2. Relationship between Vitamin D and CVD

**Figure 2:**
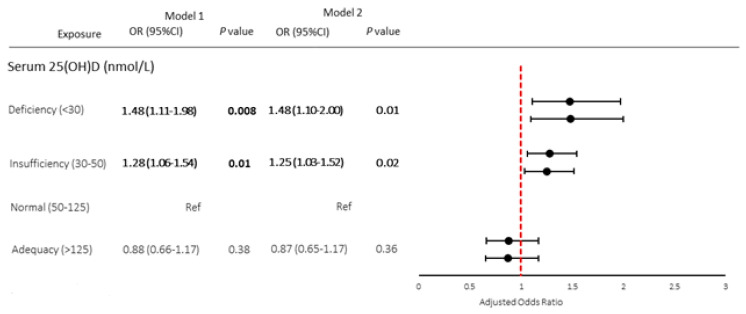
Forest plots of AOR (95% CI) of cardiovascular disease by vitamin D levels (serum 25(OH)D). Note: Bold indicates *p* < 0.05. Model 1 represents multivariate logistic regression analysis after adjustments for age, gender, and race. Model 2 represents multivariate logistic regression analysis after adjustments for variables in model 1 and BMI, hypertension, diabetes, hypercholesterolemia, and CRP.

The associations between 25(OH)D and CVD are summarized in Figure 2. Both the results from Model 1 and Model 2 were plotted side by side displaying various serum 25(OH)D levels: deficiency, insufficiency, normal, and adequacy. After controlling for age, gender, and race/ethnicity, participants with serum 25(OH)D deficiency had significantly higher odds of having CVD compared to those with normal 25(OH)D level (AOR = 1.48; 95% CI = 1.11–1.98; *p* < 0.05). Those with insufficient serum 25(OH)D were also more likely to have CVD than those with normal 25(OH)D levels (AOR = 1.28; 95% CI = 1.06–1.54; *p* < 0.05). After the adjustment of age, gender, race/ethnicity, BMI, hypertension, hypercholesterolemia, diabetes, and CRP level, 25(OH)D deficiency (AOR = 1.48; 95% CI = 1.10–2.00; *p* < 0.05) and 25(OH)D insufficiency (AOR = 1.25; 95% CI = 1.03–1.52; *p* < 0.05) remained as the independent risk factors for CVD. The risk for CVD disappeared in all models for those with 25(OH)D adequacy.

## 4. Discussion

The aim of this study was to clarify the role of vitamin D on CVD using a large nationally representative sample and to identify if levels of serum 25(OH)D > 125 nmol/L increase CVD risk. Our results found a significant association between serum 25(OH)D deficiency and CVD. We also found a significant association between serum 25(OH)D insufficiency and CVD. Finally, we found no association between CVD and adequacy (25(OH)D > 125 nmol/L), indicating that 25(OH)D > 125 nmol/L may not negatively impact CVD risk as has been seen in some of the literature.

Vitamin D is necessary for ideal health and the principal source of vitamin D is biosynthesis of sunlight through the skin, but such synthesis can contribute to negative outcomes, including increased risk for cancers, and is not always adequate for optimal levels. Thus, supplemental vitamin D is often prescribed or taken over the counter. It is concerning that among middle-aged and older adults in the U.S., almost 20% were vitamin D deficient [53], and individuals with darker pigmentation [54] were at greater risk for vitamin D deficiency [55], with data from the NHANES study showing 75% of non-Hispanic black participants were vitamin D deficient [55]. Of further concern, individuals may not know the optimal levels of vitamin D to take and may take more than the correct dose needed, leading to health problems.

Research has shown that one benefit of optimal vitamin D levels is its ability to reduce the risk of CVD. However, the literature has been mixed on the benefits of vitamin D on CVD with some showing a protective effect and others showing no association between vitamin D and CVD at all. Our results show a significant association between both deficiency and insufficient 25(OH)D and CVD, supporting the assertion that vitamin D has a role to play in CVD risk [28,56,57]. These are important findings as CVD is the number one killer of both men and women in the U.S. Understanding how vitamin D can reduce the risk for CVD morbidity and mortality may help healthcare providers in their decisions for recommending vitamin D supplements to their patients who show Vitamin D deficiency or insufficiency. This information is particularly important for those who already are at risk for CVD because of other health conditions or family history. 

There are also mixed findings on serum 25(OH)D > 125 (adequacy), with some researchers finding adequacy levels associated with increased risk of CVD and all-cause mortality [44], while others found no such association [45]. Our results support the literature that 25(OH)D adequacy does not increase CVD risk. This is an important finding for healthcare providers as they make vitamin D supplement decisions for their patients. Previously, providers have had to determine the value of increasing 25(OH)D levels for their patients to provide CVD protection by prescribing vitamin D against the cost of increased risk of CVD. Our results show that providers may be able to prescribe vitamin D without having to weigh the pros and cons.

While these findings are important, certain limitations should be noted. Data collected did not include skin pigmentation, latitude, season of the year, or temperature of the participant’s residence. Data from the study is cross-sectional rather than longitudinal; thus, interpretations should be made with caution. CVD history was also self-reported, which might be subject to recall bias and misreporting. We also did not have information on whether participants were taking vitamin D supplements, and thus we were unable to determine whether 25(OH)D levels were influenced by natural means (i.e., sunlight) as opposed to supplements. Additionally, our sample with CVD conditions was younger than the non-CVD group. It is worth noting as well that some researchers have found an association between UVA exposure and CVD risk and hypertension through the release of nitric oxide [37,38], although not all associations show this is independent of vitamin D. Future studies should continue to assess the role of vitamin D and the release of nitric oxide on CVD risk.

Despite these limitations, our study has several strengths. We used a large and nationally representative sample, which can be generalizable to the U.S. population. Our sample size is relatively larger than many studies, which allows us to draw meaningful conclusions. We also used the most recent NHANES data. This enables us to provide updates to the literature.

Future research should also explicitly include an examination of skin pigmentation, and the altitude and temperature of the participants’ residence in the U.S. While our data came strictly from the U.S., a more global examination may provide a different perspective.

## 5. Conclusions

This study suggests that maintaining optimal vitamin D levels may have potential benefits for cardiovascular health. Both vitamin D deficiency and insufficiency were associated with an increased risk of CVD, including stroke, heart disease, heart attack, and heart failure, the leading cause of death in the U.S., and excess vitamin D was not associated with increased CVD risk. Healthcare professionals should then consider vitamin D supplements for their patients who show deficiency or insufficiency, without worry about adequacy increasing CVD risk. It is important for individuals to maintain adequate vitamin D levels through exposure, dietary sources, and supplementation while considering individual health needs and consulting with healthcare professionals. Lastly, vitamin D may have implications beyond its role in cardiovascular health, including its involvement in bone health, immune regulation, and potential impact on cancer prevention and treatment. Further research is needed to fully understand the mechanisms and benefits of vitamin D in these contexts.

## Figures and Tables

**Table 1:** Demographic characteristics (NHANES 2015–2018).

Variable	Mean (Interquartile Range)	*n* (%)
Age (year)	50.3 (29.0)	
Gender		
Male		4704 (47.9)
Female		5121 (52.1)
Race/Ethnicity		
Hispanic		2672 (27.2)
Non-Hispanic White		3341 (34.0)
Non-Hispanic Black		2112 (21.5)
Other Race		1700 (17.3)
Body Mass Index	29.7 (8.8)	
Blood Pressure		
No		6194 (63.0)
Yes		3631 (37.0)
Diabetes		
No		8075 (82.2)
Yes		1750 (17.8)
Cholesterol		
No		6369 (64.8)
Yes		3456 (35.2)
C-reactive Protein		
<0.2		4937 (50.2)
≥0.2		4888 (49.8)
Vitamin D		
Deficiency		770 (7.8)
Insufficiency		2252 (22.9)
Normal		6370 (64.8)
Adequacy		433 (4.4)
Cardiovascular Disease		
No		8797 (89.5)
Yes		1028 (10.5)

**Table 2:** Comparison of the characteristics between CVD and non-CVD groups.

Variable	Non-CVD Group (*n* = 8797)	CVD Group (*n* = 1028)	Statistic	*p*-Value
Age (year), mean (interquartile range)	48.4 (29.0)	66.4 (17.0)	−42.8	**<0.001**
Gender, *n* (%)				
Male	4108 (46.7)	596 (58.0)	−0.1	**<0.001**
Female	4689 (53.3)	432 (42.0)		
Race/ethnicity, *n* (%)				
Hispanic	2475 (28.1)	197 (19.2)	0.1	**<0.001**
Non-Hispanic White	2869 (32.6)	472 (45.9)		
Non-Hispanic Black	1865 (21.2)	247 (24.0)		
Other	1588 (18.1)	112 (10.9)		
Body Mass Index (kg/m^2^) mean (interquartile range)	29.6 (8.7)	30.9 (8.8)	−5.3	**<0.001**
Blood pressure, *n* (%)				
No	5930 (67.4)	264 (25.7)	0.3	**<0.001**
Yes	2867 (32.6)	764 (74.3)		
Diabetes, *n* (%)				
No	7465 (84.9)	610 (59.3)	0.2	**<0.001**
Yes	1332 (15.1)	418 (40.7)		
Cholesterol, *n* (%)				
No	5995 (68.1)	374 (36.4)	0.2	**<0.001**
Yes	2802 (31.9)	654 (63.6)		
C-reactive protein, *n* (%)				
<0.2	4514 (51.3)	423 (41.1)	0.1	**<0.001**
≥0.2	4283 (48.7)	605 (58.9)		
Vitamin D, *n* (%)				
Deficiency	701 (8.0)	69 (6.7)	0.1	**<0.001**
Insufficiency	2062 (23.4)	190 (18.5)		
Normal	5670 (64.5)	700 (68.1)		
Adequacy	364 (4.1)	69 (6.7)		

Note: bold indicates *p* < 0.05.

## Data Availability

Data are available at https://wwwn.cdc.gov/nchs/nhanes/Default.aspx (accessed on 8 August 2023).
